# Clinical Intersections Among Idiopathic Language Disorder, Social (Pragmatic) Communication Disorder, and Attention-Deficit/Hyperactivity Disorder

**DOI:** 10.1044/2020_JSLHR-20-00050

**Published:** 2020-10-15

**Authors:** Sean M. Redmond

**Affiliations:** a Department of Communication Sciences and Disorders, University of Utah, Salt Lake City

## Abstract

**Purpose:**

Estimates of the expected co-occurrence rates of idiopathic language disorder and attention-deficit/hyperactivity disorder (ADHD) provide a confusing and inconsistent picture. Potential sources for discrepancies considered so far include measurement and ascertainment biases (Redmond, 2016a, 2016b). In this research symposium forum article, the potential impact of applying different criteria to the observed co-occurrence rate is examined through an appraisal of the literature and an empirical demonstration.

**Method:**

Eighty-five cases were selected from the Redmond, Ash, et al. (2019) study sample. Standard scores from clinical measures collected on K–3rd grade students were used to assign language impairment status, nonverbal impairment status, social (pragmatic) communication disorder status, and ADHD status. Criteria extrapolated from the specific language impairment (Stark & Tallal, 1981), developmental language disorder (Bishop et al., 2017), and *Diagnostic and Statistical Manual of Mental Disorders, Fifth Edition* language disorder (American Psychiatric Association, 2013) designations were applied.

**Results:**

The *Diagnostic and Statistical Manual of Mental Disorders, Fifth Edition* language disorder designation and its separation of language disorder from the social (pragmatic) communication disorder designation provided the clearest segregation of idiopathic language deficits from elevated ADHD symptoms, showing only a 2% co-occurrence rate. In contrast, applying the broader developmental language disorder designation raised the observed co-occurrence rate to 22.3%. The specific language impairment designation yielded an intermediate value of 16.9%.

**Conclusions:**

Co-occurrence rates varied as a function of designation adopted. The presence of pragmatic symptoms exerted a stronger influence on observed co-occurrence rates than low nonverbal abilities. Impacts on clinical management and research priorities are discussed.

**Presentation Video:**

https://doi.org/10.23641/asha.13063751

Children affected by language disorders represent a heterogeneous group. For some children, their acquisition of linguistic proficiency is complicated by either injury or concomitant neurodevelopmental disruption or both. Language outcomes within groups of children affected by clinical conditions such as strokes, traumatic brain injury, cerebral palsy, hearing impairments, autism spectrum disorder, intellectual disability (ID), and attention-deficit/hyperactivity disorder (ADHD) have been highly variable, complicating efforts to attribute children's linguistic deficits to their deficits in other areas (see Rice, 2016, for a review). Even more puzzling is that many more children with language disorders appear to have them in the absence of identifiable injury or any clinically significant deficits in other areas of development (Beitchman et al., 1986; Norbury et al., 2016; Tomblin et al., 1997). The existence of idiopathic cases as the prototypical presentation of language disorders within the pediatric population suggests that nonverbal deficits of the sort that lead to significant disruptions in other areas of development are neither necessary nor sufficient preconditions. The idiopathic profile provides a yardstick for understanding nonprototypical cases of language disorder that may differ significantly from prototypical cases in either symptom presentation, severity, or progression. The idiopathic profile of language disorder is also needed to properly attribute the contributions of additional comorbidities on individual interpersonal, academic, and vocational outcomes.

This research symposium forum article provides the companion report to my presentation at the 2019 American Speech-Language-Hearing Association's (ASHA) Convention Research Symposium *Advances in Specific Language Impairment Research and Intervention*. Specific language impairment (SLI) has been one of the more commonly used terms in research studies to refer to the idiopathic subgroup of children with language disorders (Bishop, 2014). The timing of the ASHA Convention Research Symposium was such that it occurred within the backdrop of considerable discussions among researchers and practitioners on the utility of maintaining the SLI term and its criteria to refer to cases of idiopathic language disorder (see Volkers, 2018a, 2018b). Specifically, an alternative designation, developmental language disorder (DLD), associated with more flexible criteria than customarily associated with SLI and proposed by the CATALISE international consortium of scholars and practitioners led by D. V. M. Bishop (Bishop et al., 2016. 2017), had been appearing with increasing regularity in research reports.^1^ Preceding the CATALISE recommendations, the American Psychiatric Association (APA) published the *Diagnostic and Statistical Manual of Mental Disorders, Fifth Edition* (*DSM-5*; APA, 2013) establishing new guidelines for diagnosing language disorder within the larger *DSM* taxonomy (*DSM-5* language disorder [*DSM-5-LD*]). *DSM-5* also introduced a new communication disorder, social (pragmatic) communication disorder (S(P)CD), a designation intended to be separate from language disorder and autism spectrum disorder. The primary clinical features of S(P)CD consists of persistent difficulties in communicating for social purposes, in adjusting to listener needs, in following conversational and narrative conventions, and in understanding nonliteral and ambiguous meanings.

Differential diagnosis is the linchpin of any successful clinical taxonomy, a prerequisite for the just allocation of personalized services to support those who need them when they need them. Molecular genetic studies and other empirical efforts to account for the underlying disruptions involved in disorders are highly dependent on our ability to differentiate one clinical condition from another. Reproducibility and external validity associated with these and other empirical efforts are compromised when researchers apply criteria inconsistently. Furthermore, there are serious ethical and forensic implications for practitioners when their diagnostic decisions are guided by overly permissive criteria or when they deviate too far from evidence-based practices (Harrison, 2017). Although the SLI, DLD, and *DSM-5* language disorder designations for idiopathic disorder overlap considerably, there are potentially nontrivial differences as well that could affect our capacity to differentiate idiopathic language disorder from other neurodevelopmental conditions and, in turn, could complicate efforts at identifying and treating cases of comorbidity. The main areas of divergence across the SLI, DLD, and *DSM-5* language disorder designations are the degrees to which children with concomitant low nonverbal abilities and children with pragmatic symptoms are accommodated.

One clinical boundary worth revisiting in this context of increasing diagnostic options is the separation of idiopathic language disorder from ADHD. Commonly cited estimates of the expected co-occurrence rates of idiopathic language disorder and ADHD date as far back as the 1980s (Cantwell & Baker, 1987; Gualtieri et al., 1983), and new estimates arrive in the literature at a regular clip. As a collection, however, these reports provide practitioners and researchers with a confusing and inconsistent picture. Some reports provide estimates that are comparable to diagnostic rates observed within the general population (Lindsay & Dockrell, 2008; Redmond & Rice, 2002; Rescorla et al., 2007; Whitehouse et al., 2011; Willinger et al., 2003). Other reports indicate substantial overlap between the two disorders (Gualtieri et al., 1983; Trautman et al., 1990; Walsh et al., 2013; Warr-Leeper et al., 1994). Potential sources of variability include a variety of measurement and ascertainment biases (see Redmond, 2016a, 2016b, for reviews). Variations across practitioners and researchers in their personal adherence to the SLI, DLD, or *DSM-5* language disorder criteria for idiopathic language disorder could introduce another major source of instability.

In this research symposium forum article, the extent to which differences across the SLI, DLD, and *DSM-5* language disorder designations could prejudice our understanding of the boundaries between idiopathic language disorder, and ADHD is examined through an appraisal of the relevant literature and an empirical demonstration. To illustrate potential trade-offs involved in adopting one designation over the others in observed rates of co-occurrence, cases selected from the Redmond, Ash, et al. (2019) study sample were subjected to a series of segregations according to my extrapolation of each designation's criteria. This convenience sample was well suited to this exercise, because it represents a combination of both community and clinically ascertained cases of language disorder, ADHD, and their co-occurrence. Clinical criteria involving neurodevelopmental disorders are rarely prescriptive to the extent that specific clinical instruments or cutoffs are provided. For example, the operationalization of common criteria such as “abilities below those expected for age” is deliberately left open to interpretation. This has certainly been true for idiopathic language disorder, and some readers will undoubtably have different SLI, DLD, and *DSM-5* language disorder extrapolations than those used here. Nonetheless, the absence of universal consensus on how to implement each designation's criteria does not preclude using their differences to explore the clinical intersections among idiopathic language disorder, S(P)CD, and ADHD.

## Differentiating ADHD From SLI

ADHD provides a useful test of the integrity of proposed diagnostic boundaries for idiopathic language disorder. ADHD is one of the most commonly diagnosed clinical conditions in childhood. An estimated 9.4% of children in the United States are expected to receive a diagnosis of ADHD at some point during their compulsory schooling (Danielson et al., 2018). The management of childhood ADHD involves a regimen incorporating pharmacological, behavioral, cognitive–behavioral, family, and educational interventions that would be contraindicated in non-ADHD cases (Barkley, 2006). Among practitioners and researchers, it is widely accepted that the symptoms of other clinical conditions, such as anxiety, learning disabilities, intellectual disabilities, and language disorders, can mimic ADHD symptoms (Barkley, 2006; Brock et al., 2009; Whitcomb, 2018). This is reflected in the differential diagnosis section for ADHD in the *DSM-5* where it is noted that, in academic settings, “children with specific learning disability may appear inattentive because of frustration, lack of interest, or limited ability” (APA, 2013, p. 64). Differential diagnosis of idiopathic language disorder in the context of potential ADHD requires robust measurement systems and consideration of potentially overlapping symptoms between these two common conditions.

In a series of investigations, my colleagues and I have examined the issue of overlapping clinical features from both sides. We have considered whether signs and symptoms of idiopathic language disorder could be mischaracterized during routine clinical assessments as supportive of a diagnosis of ADHD or another socioemotional behavioral disorder (Ash et al., 2017; Redmond, 2002; Redmond & Ash, 2014; Redmond & Rice, 1998, 2002; Redmond, Hannig, & Wilder, 2019). We have also looked into the possibility that poor performance on language measures could potentially reflect deficits in children's attention, hyperactivity, or impulsivity rather than result from underlying linguistic deficits (Redmond, 2004, 2005; Redmond et al., 2015, 2011). Our investigations of clinical measures and their potential biases were guided by the SLI phenotype (Leonard, 2014; Stark & Tallal, 1981; Tager-Flusberg & Cooper, 1999).

Criteria for SLI status have varied across research studies, but a common formulation, and the one implemented across our investigations, has been to define SLI as the presence of linguistic abilities significantly below age expectations in the absence of clinically significant deficits in other domains. The operationalization of the inclusionary aspects of this formulation includes performance on language composite scores from an omnibus standardized language test (e.g., Clinical Evaluation of Language Fundamentals; Semel et al., 2003) below a standard score of 85. The rationale for selecting language composites rather than tests/subtests targeting only one linguistic domain has been to accommodate for variability across children in their presenting symptoms across expressive and receptive language skills (Stark & Tallal, 1981). The exclusionary aspects of the formulation or documentation of absence of clinically significant deficits in other areas have usually included performance within normal limits on a standardized nonverbal test after taking the standard error of measurement into account. In other words, to be included within the group with SLI in group comparison studies, potential participants need to demonstrate nonverbal standard scores at or above 80. Normal hearing acuity is often confirmed in research studies by requiring potential participants to pass hearing screenings at conventional levels. Finally, confirmation via parental report that a potential participant has a negative history of brain injury and has not received a diagnosis of autism or other neurodevelopmental disorders is typically required (see Leonard, 2014).

Standardized behavioral rating scales, like the widely used Child Behavioral Checklist (CBCL; Achenbach & Rescorla, 2001), represent the most efficient method for identifying ADHD and other socioemotional behavioral disorders in children and are widely used to estimate prevalence rates and document treatment effects (Brock et al., 2009; Sayal et al., 2018; Whitcomb, 2018). For the routine evaluation of children with either known or suspected language disorders, rating scales would also be preferred over alternatives such as projective–expressive techniques, sociometric measures, self-reports, continuous performance tests, or executive function measures because these methods have well-documented psychometric limitations that are probably compounded when applied to children with limited verbal abilities (Nyongesa et al., 2019; Riccio et al., 2001; Webster et al., 1999; Whitcomb, 2018). Even so, Redmond (2002) reviewed five standardized behavioral rating scales and identified several aspects of their design that made them prone to mischaracterize language impairments as ADHD and other socioemotional behavioral disorders. Recently, Redmond, Hannig, and Wilder (2019) revisited newer editions of the scales reviewed by Redmond (2002). We expanded our audit to include newer scales that have been appearing with regularity in research and clinical reports. Improvements in behavioral rating scale design over the 17-year interim were noted in key areas. These included expanded representation of children with language disorders in the standardization samples of rating scales, disaggregated norms for children with learning disabilities, and procedures for identifying inordinately punitive ratings. Unfortunately, items that could be considered symptomatic of language disorders or their academic consequences (e.g., “speech problems,” “difficulty following directions,” “difficulty completing assignments,” “listens carefully”) still appeared frequently within the inventories of standardized behavioral rating scales. Redmond and Ash (2014) examined the effect of removing language and academic items on the diagnostic accuracies of two commonly used instruments, the CBCL and the Conners' Parent Rating Scale–Revised (CPRS-R: Conners, 2004). More adjustments were required of the instruments' attention/inattention, social problems, and internalizing subscales than externalizing, hyperactivity, or other subscales. More items from the CPRS-R's inventory than the CBCL's were affected. Notably, the CBCL DSM ADHD subscale did not require adjustment, whereas the CPRS-R ADHD subscale did. Results indicated that removal of language and academic items across the different behavioral subscales involved improved their specificity for discriminating cases of ADHD from SLI (area under the response operating characteristic curve [AUC] range for adjusted scores: .79–.97) with very little impact on their sensitivity to separate cases of ADHD from typical development (AUC range for adjusted scores: .83–1.00).

Differential diagnosis is a coin with two sides. It is equally important to consider the potential impact ADHD status might have on the valid assessment of children's language skills. For example, when children display signs of inattention and distractibility over the course of a formal language assessment it can be unclear how much faith should be put into the integrity of the information collected. Fortunately, signs of language disorder identified through language sample analysis (LSA) do not require assumptions that children were consistently paying attention to the relevant aspects of the testing prompts or that they understood the task. This makes LSA a particularly apt starting point to consider the potential impact of ADHD on children's language skills. Redmond (2004) collected 30-min conversational samples collected during free-play from children with ADHD, SLI, and typical development (TD). Results indicated that only the SLI group presented with semantic and grammatical deficits relative to the TD group, as indexed by the number of different words, the mean length of utterance, and a composite measure of their proficiencies with marking tense in obligatory contexts. Despite its many virtues, however, LSA does have limitations that curb widespread use among practitioners. Chief among these are the expertise, time, and resources involved in collecting, transcribing, and analyzing language samples relative to other clinical measures. In contrast, sentence recall and elicited tense-marking measures require considerably less expertise and can be administered and interpreted relatively quickly. Most importantly, these measures have been shown across several studies to successfully discriminate cases of SLI from cases of typical development (see Pawłowska, 2014). Sentence recall and tense-marking measures were collected from the same sample of children in the study of Redmond (2004). Results indicated that children in the ADHD group performed similarly to children in the TD group on these language tasks and at a considerably higher level of accuracy than children in the SLI group (Redmond, 2005). Redmond et al. (2011) replicated these results in a larger study sample and extended their coverage to include nonword repetition and narrative measures as well. In each area, children with ADHD performed similarly to their peers with typical development, whereas robust linguistic deficits were associated with SLI status. Areas under the receiver operating characteristic curve differentiating ADHD from SLI using language measures ranged from .88 to .96. Our pattern of group differences across language measures were replicated by Parigger (2012) in a study sample of 67 Dutch-speaking children.

In summary, when clinical measures are selected carefully and, where appropriate, are adjusted to account for potential overlapping symptoms, differential diagnosis of SLI and ADHD and the identification of their comorbidity can be successfully executed. However, by design, the studies addressing these issues did not include children with concomitant low nonverbal abilities or children who presented with pragmatic deficits only. The presence of concomitant low nonverbal abilities in children with more broadly defined language disorders relative to children with SLI-type profiles has been associated with lower levels of language performance, slower language growth, and higher levels of risk for emotional and behavioral disorders (Beitchman et al., 1989; Law et al., 2009; Rice et al., 2004; Snowling et al., 2006; Wilder & Redmond, 2020). Thus, the extent to which the newer designations for idiopathic language disorder DLD and *DSM-5* language disorder would enjoy the same level of taxonomic clarity observed with SLI and ADHD is unknown.

## DLD

The expression “developmental language disorders” was originally offered as a superordinate designation by researchers to refer to a broad collection of neurodevelopmental conditions involving disrupted language acquisition, including SLI, Down syndrome, Williams syndrome, autism, fragile X, cerebral palsy, and dyslexia (e.g., Kamhi et al., 2007; Norbury et al., 2008; Rice & Warren, 2004; Verhoeven & van Balkom, 2004). Over the course of the CATALISE Delphi exercise, the DLD term was repurposed by the consortium to only refer to the subgroup representing idiopathic language disorder. The term “language disorder” was offered as a replacement for the collection of clinical conditions previously identified by the DLD designation. The major difference between the newer DLD designation and the SLI designation that preceded it is the inclusion of children with low nonverbal abilities within its catchment. Although both SLI and DLD criteria exclude frank cases of ID associated with biomedical syndromes and conditions, the DLD designation deliberately includes children the SLI designation would not. These children represent the subgroup that presides in the potentially diagnostically indeterminate areas between ID and SLI and between ID and learning disability (i.e., children with both verbal and nonverbal abilities between 1.0 and 2.0 *SD*s below age expectations). Although a relatively understudied subgroup of children, prevalence estimates suggest that children with this profile represent 3.4%–6.9% of the student population (Beitchman et al., 1986; Tomblin et al., 1997). Some research groups have used the designation “nonspecific language impairment” (NLI) to differentiate this subgroup from the SLI subgroup when studying language and academic outcomes (Ellis Weismer et al., 2000; Fey et al., 2004; Rice et al., 2004). Outside speech-language pathology, children with this profile have been variously described by clinical and school psychologists, as affected with global delay, mild mental retardation, borderline mental retardation, borderline intellectual functioning, and learning disability (MacMillan & Siperstein, 2002; Peltopuro et al., 2014; Wieland & Zitman, 2016).

The rationale provided by the CATALISE group for expanding the phenotype of idiopathic language disorder was multifaceted. It included the logistical challenges facing practitioners for confirming discrepancies between verbal and nonverbal abilities as well as empirical observations of similar levels of response to language treatments across children with various nonverbal abilities. With its expanded catchment, the new DLD designation also aligned better than the SLI designation did with the highly influential model of language disorders provided by Bloom and Lahey (1978) and its various manifestations, including the definition of “spoken language disorder” currently offered by ASHA.^2^ However, there is one exception worth noting. Special effort was made by the CATALISE group to align its designation with the DSM taxonomy and to differentiate language deficits attributable to DLD from language deficits associated with autism spectrum disorder. In this regard, the DLD and SLI designations share a common point of departure from the more etiologically neutral models of language disorder of Bloom and Lahey and ASHA.

## Language Disorder in the DSM-5

The *DSM-5* offers a comprehensive taxonomy intended to cover the full range of neurodevelopmental and psychiatric conditions across the life span. This includes the clinical designation of language disorder, situated within the communication disorders section of neurodevelopmental disorders along with speech sound disorder, childhood-onset fluency disorder, and S(P)CD. As an integrated scheme, adjustments that occur within one clinical designation across editions of the DSM taxonomy have both intended and unintended consequences on other clinical designations. For example, major adjustments in the criteria for autism spectrum disorders occurred over the transition from *Diagnostic and Statistical Manual of Mental Disorders, 4th Edition Text Revision *(*DSM-IV*; American Psychiatric Association, 2000) TR to *DSM-5* involving the role of language impairments. In the earlier scheme, language impairments represented a required element of the diagnosis of autism (and other pervasive developmental disorders), whereas in the current *DSM-5* taxonomy, language impairment is no longer a required element for diagnosis but instead represents a potential specifier for autism spectrum disorder. These adjustments resulted in fewer individuals meeting *DSM-5* criteria relative to *DSM-IV* TR criteria (Taheri & Perry, 2012; Young & Rodi, 2014). Some of the individuals with profiles consistent with a *DSM-IV* TR autism/pervasive developmental disorder designation would align better with the new *DSM-5* S(P)CD designation, which shares some of the social communication features with autism spectrum disorder but is differentiated from it by the severity/scope of these difficulties and by the absence of restricted, repetitive patterns of behavior, interests, and activities. The criteria for S(P)CD stipulates further that it represents a condition separate from language disorder.

Other adjustments in the *DSM-5* taxonomy relevant to considerations of idiopathic language disorder include historical changes replacing the mental retardation designation with the ID designation (Tassé, 2016). Across editions of the DSM, intelligence testing criteria for identifying clinically significant deficits in individuals' intellectual functioning have paradoxically become both increasingly more stringent and yet more flexible. For example, subaverage IQ performance associated with ID has been described in the *DSM-5* as characteristically aligned with levels starting at 2 *SD*s below age expectations (standard scores: 65–70; APA, 2013), whereas in earlier editions, subaverage performance began at 1 *SD* below age expectation. Conversely, *DSM-5* also recognizes that ID can occur at higher levels of IQ performance if individuals present with significant deficits in their adaptive functioning/independence. However, one challenge to applying the qualitative descriptors of adaptive functioning offered by the *DSM-5* to the task of differentiating language disorder from ID is that many elements within the Conceptual and Social Domains associated with *DSM-5* mild and moderate ID designations are based on language performance (“language and pre-academic skills develop slowly,” “spoken language is much less complex than that of peers,” and “communication, conversation, and language are more concrete and immature than expected for age”; APA, 2013, p. 35). This is exacerbated further by the natural consequences of language disorder that inevitably negatively impact on affected individuals' social and academic adaptive functioning. Thus, in practical terms, preventing language disorder from becoming synonymous with mild/moderate ID by default requires nonverbal IQ testing of some kind demonstrating some threshold level of performance. The consequences of applying different values to this threshold warrants additional investigation.

## Criteria and Their Consequences

The SLI, DLD, and *DSM-5* designations are differentiated by the extent to which profiles indicative of low nonverbal abilities and social communication deficits are either excluded from or incorporated into their formulations. SLI criteria exclude cases with below normal nonverbal abilities/borderline intellectual functioning within its catchment but include cases with co-occurring social communication deficits. In contrast, the *DSM-5* criteria for language disorder exclude children whose difficulties would be better captured by its S(P)CD designation, but they would include cases with co-occurring low nonverbal abilities/borderline intellectual functioning. More specifically, the *DSM-5* criteria combine cases that meet SLI criteria with cases of concomitant nonverbal abilities that are below normal but do not meet suggested thresholds for ID (IQ standard score < 70). The CATALISE DLD designation would include all cases meeting S(P)CD criteria, with or without accompanying deficits in other areas of language. Like the *DSM-5*, DLD criteria also incorporates cases of low nonverbal abilities/borderline intellectual functioning down to the current thresholds of ID, provided either S(P)CD-type deficits or deficits in other language areas were present. Although hotly contested (see Volkers, 2018a, 2018b), it is unclear whether the distinctions that have been brought in by these different designations make a practical difference to differential diagnosis.

To examine the taxonomic consequences of applying different criteria, I utilize cases drawn from a study sample of early elementary students associated with one of our recent projects (see Redmond, Ash, et al., 2019). This study sample combined community-based and clinically based ascertainment procedures, ensuring that both undiagnosed and misdiagnosed cases were represented. Students were recruited from the community into the study through a series of school-based screenings. To supplement the community sample and increase the number of potential cases of language disorder, students were also recruited from the caseloads of certified speech-language pathologists working in different school districts and clinics.

## Method

### The Redmond, Ash, et al. (2019) Study Sample

School-based language screenings involving 1,060 K–3rd grade students over a 4-year period were conducted to identify students at risk for language impairments. Students enrolled in regular education and students receiving speech-language, emotional–behavioral, reading, or learning disability services participated. A supplemental group of 58 students receiving services for language impairments recruited from the caseloads of certified speech-language pathologists working in different school districts and clinics were also screened. Data from three of these 58 students were not included in the Redmond, Ash, et al. (2019) screening study analyses and were likewise excluded from the current analysis. One student presented with minimal verbal abilities and failed our language screenings but was excluded because, during confirmatory testing, the student was unable to complete our nonverbal and phonological assessments. Two additional students receiving clinical services who participated in our screenings were not included because they were older than our age limit of 10;6 (years;months). Out of the remaining 55 students referred from practitioner caseloads, 21 passed our screenings.

A subset of students from both the community and clinical samples, representing those who had failed the screenings and a random sampling of those who had passed (determined by lottery) were invited to participate in laboratory-based confirmatory assessments. Examiners conducting the confirmatory assessments were naïve to children's screening performance and to any clinical services these children were receiving at the time of testing (see Redmond, Ash, et al., 2019, for further details).

### Operational Definitions of Language Impairment, Nonverbal Impairment, ADHD, and S(P)CD

Eighty-five cases from the Redmond, Ash, et al. (2019) study sample of the 251 who had completed confirmatory testing represent the convenience sample used here to examine co-occurrence rates. Participant characteristics are presented in Table 1. Cases were selected for analysis because they meet one or more of our experimental criteria for idiopathic language disorder, S(P)CD, and ADHD (details presented below). Consistent with the exclusionary criteria shared across the SLI, DLD, and *DSM-5* language disorder designations, potential participants identified by school records and/or parental report with brain injury, autism spectrum disorder, or ID were excluded. However, even with these neurodevelopmental restrictions in place, the range of nonverbal cognitive performance associated with the convenience sample was considerable. Children with both very low and very high estimated nonverbal abilities were included. All participants providing data were monolingual speakers of English. As part of the confirmatory testing protocol used in the screening study, all participants passed hearing and speech screenings. Families of children being treated with behavioral medications were instructed to suspend these medications for 24 hr prior to confirmatory behavioral testing.

**Table 1.** Participant characteristics (*N* = 85): means, standard deviations (in parentheses), and ranges (in italic).

Age	% Male	Ethnicity and race	Maternal education	CELF-4	NNAT	ADHD	PC5
8;0 (1;2) *6;10–10;3*	65.9	**Ethnicity:** Hispanic = 10.6% Non-Hispanic = 89.4% **Race:** Am. Ind. = 1.2% Asian = 4.7% Black = 4.7% Pac. Isl. = 2.4% White = 87.0%	3.23 (0.93) *1–5*	79.91 (19.98) *40–132*	100.72 (16.00) *73–151*	60.64 (9.03) *50–77*	81.72 (15.62) *43–119*

*Note.* Age is in years;months. Ethnicity/race categories are based on U.S. Census; Am Ind = American Indian; Pac Isl. = Pacific Islander. Maternal education: 1 = some high school, 2 = high school diploma/General Educational Development, 3 = some college, 4 = 4-year college degree, 5 = some graduate school/advanced degree. CELF-4 = Clinical Evaluation of Language Fundamentals–Fourth Edition core language standard score (*M* = 100, *SD* =15); NNAT = Naglieri Nonverbal Ability Test standard score (*M* = 100, *SD* = 15); ADHD = Child Behavioral Checklist Attention-Deficit/Hyperactivity Disorder *Diagnostic and Statistical Manual of Mental Disorders* subscale *T* score (*M* = 50, *SD* = 10), a higher score indicates elevated levels of parental concern; PC5 = standardized pragmatic composite of the following five subscales on the Children's Communication Checklist–Second Edition: Coherence, Initiation, Scripted Language, Context and Nonverbal Communication (*M* = 100, *SD* =15).

For this analysis, children with a core language standard score on the Clinical Evaluation of Language Fundamentals–Fourth Edition (CELF-4; Semel et al., 2003) of ≤ 85 were assigned *“*language impairment” status, and those with a Naglieri Nonverbal Ability Test (NNAT; Naglieri, 2003) standard score of ≤ 80 were assigned “nonverbal impairment” status. These test scores were used further to place cases into either the SLI or NLI groups, following Tomblin et al. (1997). The NLI designation used by Tomblin and colleagues is homologous to the borderline intellectual functioning designation (Wieland & Zitman, 2016). Following clinical cutoffs suggested by the instrument, a CBCL *DSM-5* ADHD syndrome criteria *T* score of ≥ 65 was used to indicate children's ADHD status (Achenbach & Rescorla, 2001). A pragmatic composite (PC5) standard score of ≤ 80 based on parental ratings for five subscales from the Children's Communication Checklist–Second Edition (CCC-2; Bishop, 2006) provided an estimate of children's S(P)CD status (Coherence, Initiation, Scripted Language, Context, Nonverbal Communication). Ash et al. (2017) selected these subscales as a proxy measure of S(P)CD based on their audit of the CCC-2's coverage of *DSM-5*–type symptoms. The Social Relations and Interests subscales designed to screen for potential symptoms of autism spectrum disorders were not included in the PC5 measure.

## Results

**Figure 1.**
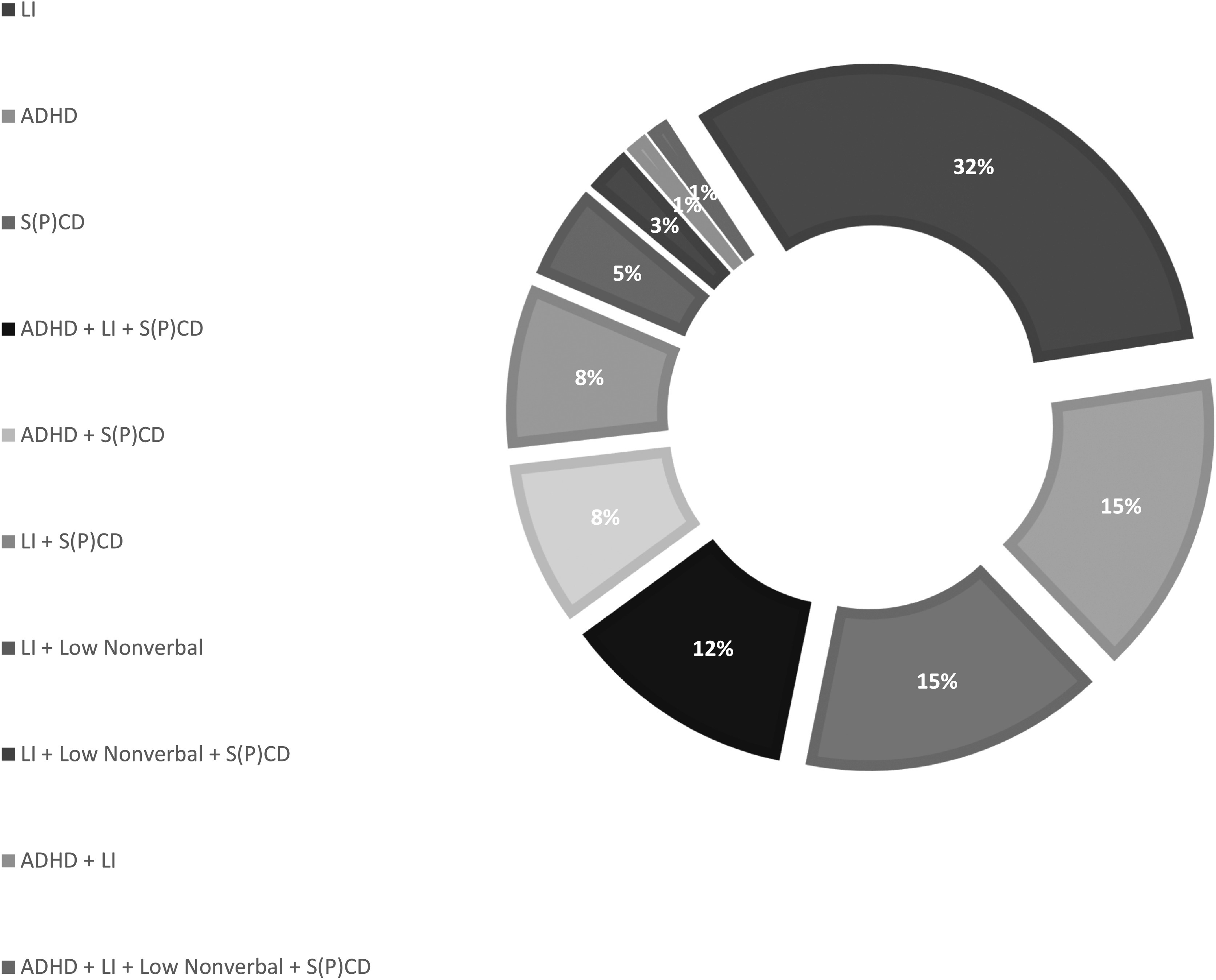
Proportions of cases from Redmond, Ash, et al. (2019; *N* = 85) meeting criteria for one or more of the following: attention-deficit/hyperactivity disorder (ADHD), language impairment (LI), nonverbal impairment, or social (pragmatic) communication disorder (S(P)CD). This figure hides the nested and intersectional nature of the data presented more explicitly in Table 2.

Figure 1 displays the proportional outcomes of assigning clinical status to cases within the convenience sample using the CBCL, CELF-4, NNAT, and CCC-2 criteria. In other words, Figure 1 provides, in ranked order moving clockwise, the relative contributions of different profile types to the total number of cases within the convenience sample representing either language impairment, ADHD, or S(P)CD on their own, in various combinations with each other, and in combination with low nonverbal ability. From this vantage point, the prominence of noncomorbid cases (language impairment [LI] only, ADHD only, and S(P)CD only) relative to comorbid cases is visible. It is also clear when looking at the data in this manner that not all possible combinations were represented. Although there was one case that met all our clinical criteria, none of the children in the convenience sample had profiles representing ADHD + Low Nonverbal Ability, ADHD + LI + Low Nonverbal Ability, or S(P)CD + Low Nonverbal Ability. Unfortunately, Figure 1 obscures the nested and intersectional nature of the data that would be important to (re)framing co-occurrences across different designations.

Table 2 presents the consequences of placing the 85 cases into a series of nested differentiations. Each cell in Table 2 is labeled alphabetically for ease of reference. Starting with the top row, where cases were first segregated into children from the convenience sample who either met our criteria for ADHD status (indicated with a “+” in the cell) or did not meet it (indicated with a “−”), each subsequent row or “GATE” below the first one provides the results of dividing the preceding divisions further. For example, GATE 2, represented by cells C through F, shows the results of taking the first division of cases by ADHD status (+ or −; represented by cells A and B) and dividing these two cells further based on + or − CELF-4 standard score of ≤ 85, yielding four cells (+ADHD +CELF-4, +ADHD −CELF-4, −ADHD +CELF-4, and −ADHD −CELF-4). Following the sequential logic associated with Table 2 to its conclusion, cell O then identifies the single case in the sample that met all clinical criteria considered: +ADHD, +CELF-4, +NNAT, +PC5. Cell DD confirms that there were not any children selected from the Redmond, Ash, et al. (2019) study sample who did not meet at least one of our ADHD, LI, or S(P)CD criteria.

**Table 2.**
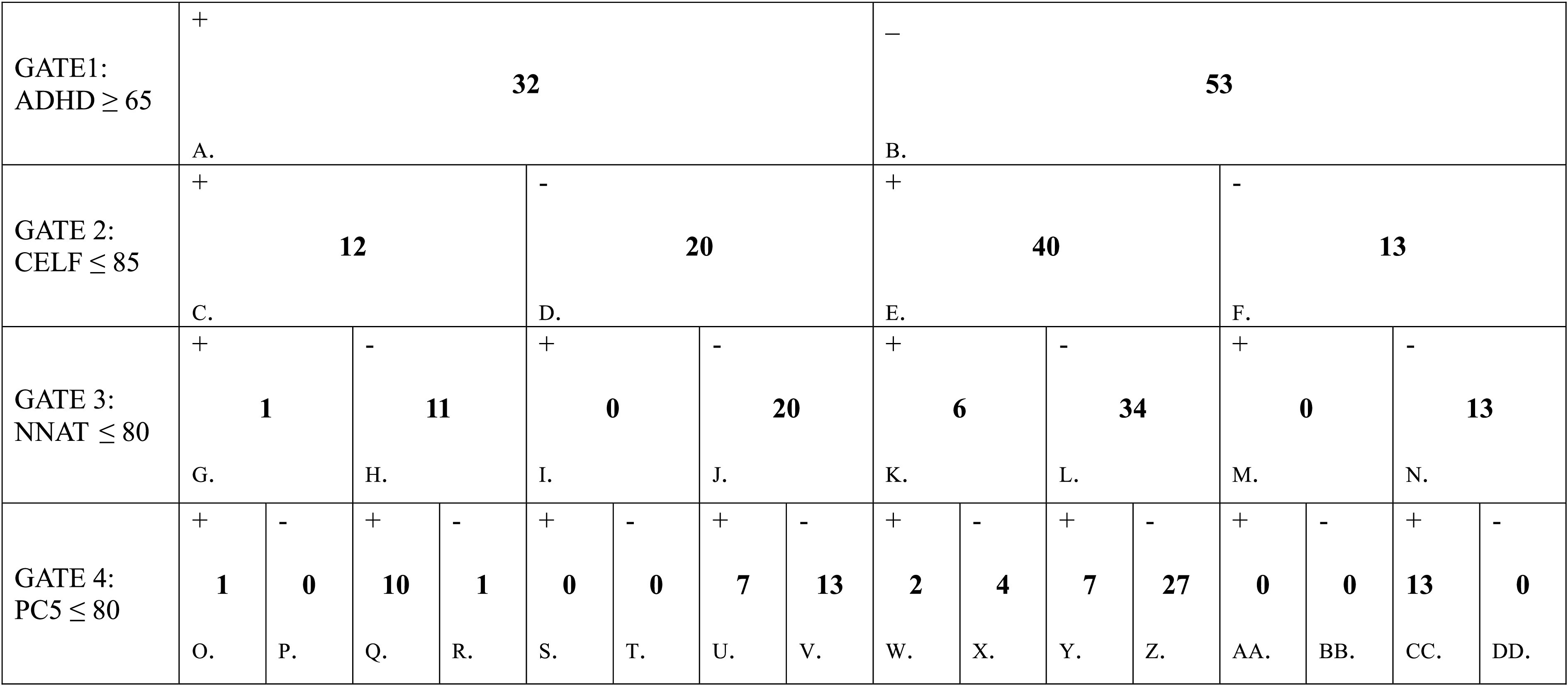
Cases from Redmond, Ash, et al. (2019; *N* = 85) meeting criteria for one or more of the following: attention-deficit/hyperactivity disorder (ADHD), language impairment, nonverbal impairment, or social (pragmatic) communication disorder.

*Note.*  + = criteria for GATE met; − = criteria for GATE not met; ADHD ≥ 65= Attention-Deficit/Hyperactivity Disorder *Diagnostic and Statistical Manual of Mental Disorders* subscale *T* score from the Child Behavioral Checklist; CELF ≤ 85 = Core Language standard score from the Clinical Evaluation of Language Fundamentals*–*Fourth Edition; NNAT ≤ 80 = standard score from the Naglieri Nonverbal Ability Test; PC5 ≤ 80 = Pragmatic composite calculated from five subtests on the Children's Communication Checklist–Second Edition following Ash et al. (2017).

Thirty-two children from the Redmond, Ash, et al. (2019) study sample met our ADHD criteria based on their CBCL DSM ADHD subscale behavioral ratings (cell A). Nineteen (59%) of these children also met criteria for either one of our variants of language impairment (SLI or NLI; cells G and H, respectively), S(P)CD (cell U), or both (cells O + Q). All but one of these 19 cases of co-occurring ADHD (cell R) also met S(P)CD criteria, based on the PC5 composite from the CCC-2. In other words, considerable overlap between pragmatics and attention/hyperactivity deficits was present in the convenience sample when co-occurrence rates of idiopathic language disorder and ADHD were based on the total number of observed cases of ADHD.

Fifty-two children from the convenience sample met CELF-4 criteria for language impairment (cells C + E). Twelve of these cases (cell C) met CBCL criteria for ADHD (23%). Most of the cases that met criteria for language impairment (45/52, 86.5%) did so within the context of measured estimates of their nonverbal abilities within the normal range (cells H + L). Eleven of the 45 children with a profile consistent with an SLI designation (24.4%) received behavioral ratings from their parents that were consistent with ADHD status (cell H). Eight of the children with SLI-type profiles (17.8%) met our criteria for S(P)CD (cells R + Y).

Seven of the 52 children with language impairment (13.5%) met our criteria for NLI—namely, below-average performance (< 80) on the NNAT (cells G + K). One of these seven children with an NLI profile (14.2%) met our criteria for ADHD (cell G). Three children with NLI profiles met our criteria for S(P)CD (42%; cells O + W). Thus, relative to rates associated with SLI, NLI was co-occurring more frequently with pragmatic limitations and co-occurring with elevated ADHD symptoms at a comparable level. These group comparisons, however, are limited by the small number of observed NLI cases.

There were 40 children who met our criteria for S(P)CD (cells O + Q + S + U + W + Y + AA + CC). Eighteen of these children (45%) also met out criteria for ADHD (cells O + Q + S + U). Twelve of these children (30%) presented with a profile consistent with an SLI designation (cells Q + W)—but most of those (10/12) were accounted for by co-occurring ADHD + SLI (cell Q; 83.33%). Three of the 40 children (7.5%) with elevated S(P)CD symptoms presented with low nonverbal abilities (cells O + S + W + AA). Thirteen of the 40 children (32.5%) with elevated S(P)CD symptoms represented cases of “S(P)CD only.” In other words, roughly a third of children assigned S(P)CD status did not meet our thresholds for assigning ADHD status and displayed normal levels of general verbal and nonverbal functioning as indexed by their CELF-4 and NNAT scores.

### ADHD Co-occurrence Rates Based on DLD, DSM-5 Language Disorder, and SLI Criteria

Co-occurrence rates were recalculated by pooling all cases that met our criteria for ADHD status with those that met criteria extrapolated from each of three different designations of idiopathic language disorder (DLD, *DSM-5* language disorder, and SLI). Then, for each designation, the number of cases that met both was divided by the total number of cases that met either. For example, the co-occurrence rate of DLD and ADHD was calculated by taking the number of comorbid cases and dividing that value by the sum of all cases of DLD and all cases of ADHD: DLD-with-ADHD / (DLD-with-ADHD + ADHD only + DLD only).

The broadest view of idiopathic language disorder providing researchers and clinicians with a potential reference to base estimations of ADHD co-occurrence rates on would be one that includes all cases in Table 2 that met either CELF-4 or PC5 criteria. This is calculated by summing C + E + S + U + AA + CC = 72. Bloom and Lahey's (1978) highly influential model of child language disorders and the etiologically neutral definition of spoken language disorder currently provided by ASHA align with this inclusive definition. The DLD designation suggested by the CATALISE group would likewise treat deficits captured by poor performances on either the CELF-4 or the PC5 as instances of the broader DLD phenotype. From this amalgamative perspective, the number of cases of co-occurring DLD and ADHD would be the sum of cells C + S + U = 19. The 13 cases of “ADHD only” appears in cell V. Cases of “DLD only” would be the sum of cells E + CC = 53. Using these values, the co-occurrence rate of DLD and ADHD is 19 / (19 + 13 + 53) = 22.35%.

In contrast, the *DSM-5* taxonomy separates language disorder from S(P)CD. This separation of linguistic domains parallels clinical traditions of distinguishing speech sound disorders from language disorders for the purposes of clinical management, even though linguistic theories and psycholinguistic models consider phonology to be a primary construct that interacts in important ways with other language constructs. To incorporate this separation in our calculation of *DSM-5* language disorder cases, we combine cells P + R + X + Z = 32. When we exclude children with significant pragmatic deficits from consideration, the single case of co-occurring *DSM-5* language disorder and ADHD without S(P)CD appears in cell R. This leaves the number of “*DSM-5* language disorder only” cases at 31. The 13 cases of “ADHD only” that do not include elevated pragmatic symptoms are represented in cell V. Using these values, the co-occurrence rate of *DSM-5* language disorder and ADHD is quite modest: 1 / (1 + 31 + 13) = 2.22%.

Criteria for SLI have been used for decades in research studies to address questions regarding the scope, progression, and underlying nature of language disorder in children when confounding nonlinguistic conditions are controlled. Children with co-occurring speech sound disorders are usually (but not always) excluded from study samples of SLI as well (see Leonard, 2014). In contrast, co-occurring pragmatic deficits have rarely been screened (but see Botting & Conti-Ramsden, 2003, for an exception). In Table 1, GATE 3 provides the results of segregating the convenience sample based on estimates of children's nonverbal abilities. The 45 cases of SLI are represented in cells H and L. The prominence of the SLI-type profile relative to the NLI-type and S(P)CD-type profiles within the convenience sample is consistent with previous epidemiological reports documenting SLI as the prototypical presentation of language disorder in children. Eleven of the SLI cases presented with concomitant SLI and ADHD (cell H), and the remaining 34 fit the “SLI-only” designation (cell L). There were 20 cases of “ADHD only” (cell J). Using these values, the co-occurrence rate of SLI and ADHD is then 11 / (11+34+20) = 16.9%.

## Discussion

Shifting enthusiasm for new terms and new criteria for idiopathic language disorder places the field of child language disorders in an interesting and potentially vulnerable place. As laid out by the CATALISE group, there are several reasons to prefer a term over its alternatives (Bishop et al., 2017). In this report, I shifted the focus on the potential value of different designations away from debates of discrepancy formulas, policy barriers to access, or the relative successes designations enjoy in catching the public's attention (Ebbels, 2014; Kamhi, 2004; Schuele & Hadley, 1999; Volkers, 2018a, 2018b) to what these designations might bring to the issues of differential diagnosis and taxonomic clarity. Clinical classification systems address our needs to structure decision making, enhance agreement across professionals, provide some measure of objectivity, and ensure reproducibility. One reason to prefer a proposed clinical term and its criteria over other options is the extent to which the designation provides reasonable boundaries with other clinical designations. What are the relative tradeoffs of adopting different designations for idiopathic language disorder?

Three different designations for idiopathic language disorder enjoying currency within the research literature and clinical practice were examined to address this question: (a) *language disorder* as defined by the *DSM-5* taxonomy (*DSM-5* language disorder), which introduced S(P)CD as a separate designation for pragmatic symptoms but allows for the inclusion of cases with accompanying low nonverbal abilities into its language disorder designation; (b) SLI, which allows for social/pragmatic symptoms when they are concomitant with semantic, syntactic, and/or verbal memory symptoms but excludes cases with low nonverbal abilities; and (c) DLD, which accommodates both social/pragmatic symptoms and low nonverbal abilities in its designation. Eighty-five cases from the Redmond, Ash, et al. (2019) community-based study sample were selected because they had at least one of the following: significantly poor performance on an omnibus language test, elevated parent-reported ADHD symptoms, or elevated parent-reported S(P)CD symptoms.

Observed co-occurrence rates within the convenience sample varied dramatically based on criteria used. For the purposes of establishing co-occurrence rates for idiopathic language disorder and ADHD, the *DSM-5* language disorder designation appears to be preferred over the others considered in this analysis. The *DSM-5* taxonomy, which separates profiles involving syntactic, semantic, and/or verbal memory deficits from profiles involving pragmatic deficits, displayed minimal (2%) overlap with cases of ADHD. In contrast, a 10-fold increase in relative co-occurrence rate was observed with the more inclusive DLD criteria, where pragmatic deficits represent one of the many possible manifestations of the DLD phenotype (see also the models of language disorder provided by Bloom & Lahey and ASHA). The SLI criteria, which exclude cases of concomitant low nonverbal ability (i.e., NLI/ borderline intellectual functioning), arrived at an intermediate value of 16.9% co-occurrence with ADHD.

The amplification of ADHD co-occurrence rates brought in by the inclusions of pragmatic deficits and, to a lesser extent, low nonverbal abilities potentially impacts other aspects of research practices in child language disorders. For example, future studies using the SLI designation to explore the socioemotional development of affected children should consider the potential explanatory value that pragmatic deficits and their links to ADHD might have on observed differences between groups and the variability observed within groups. Depending on the nature of the research questions being asked, this can be accomplished through the selection of participants that do not bias toward supporting the hypothesis (e.g., screening for pragmatic deficits/ADHD when the aim is to test potential links between verbal memory limitations and externalizing behavior problems) or through statistical modeling (i.e., controlling for the contributions of pragmatics and ADHD through covariation and mediator/moderator analyses). These considerations should apply as well to studies employing the DLD term but applying the SLI criteria (nonverbal IQ > 80) to their study samples. For studies embracing the broader DLD phenotype (nonverbal IQ > 70), the potential influence of nonverbal deficits on socioemotional development should be considered as well.

As currently conceived, the DLD criteria could be adjusted to accommodate for what appear to be rather robust qualitative differences between the S(P)CD profile and other profiles of linguistic deficit when it comes to risk for ADHD and possibly other clinical conditions. For example, a “primarily pragmatic” subtype of DLD, alongside other DLD subtypes, could capture this clinically relevant distinction. The CATALISE group failed to reach consensus on the issue of subtyping, but this is the direction the upcoming International Classification of Diseases (ICD-11) system seems to be embracing.^3^

More radical adjustments to the symptomology landscape than subtyping could be considered as well that account for the high levels of overlap observed between S(P)CD and ADHD. Pragmatic deficits of the kind presently captured by the *DSM-5*'s S(P)CD designation might be better conceptualized as a collection of symptoms that cut across various clinical designations rather than as a separate, cohesive, diagnostic entity. This would make the scope of pragmatic deficits comparable to executive function deficits and soft neurological signs that have both been characterized as being highly “transdiagnostic” (Fellick et al., 2001; Sauer-Zavala et al., 2017; Snyder et al., 2019). Transdiagnostic clinical features represent common deficits and liabilities that appear across disparate clinical conditions and, for this reason, are not particularly helpful when practitioners are engaged in differential diagnosis or trying to establish comorbidity. Transdiagnostic features, however, can be helpful during the development of individualized treatment plans (Sauer-Zavala et al., 2017).

The transdiagnostic perspective of S(P)CD-like symptoms probably aligns better than the *DSM-5*'s categorical perspective with prominent theoretical accounts. For example, Perkins' (2007) model of pragmatic impairments frames them as an emergent property of compromised communication that develops in the context of a variety of deficits (perceptual, motoric, cognitive, social development). According to Perkins, pragmatic impairments are expected to be present in any situation where synchrony across neurodevelopmental systems has been compromised for a significant amount of time. Similarly, Adams (2003) synthesis model of pragmatic impairment intervention encourages practitioners to address social, cognitive, and linguistic contributors to pragmatic disorder within their treatment plans. Investigations into pragmatic treatment approaches across groups of children affected by a variety of clinical conditions might uncover underlying mechanisms responsible for the appearance or maintenance of shared pragmatic deficits. Examples of factors that cut across clinical conditions that are potentially amenable to pragmatics-based intervention are the experiences of prolonged peer neglect and victimization (see Redmond, 2011). These and other speculations about how best to accommodate pragmatic deficits of the type captured in the *DSM-5* by its S(P)CD designation within the larger contexts of idiopathic language disorder, ADHD, and other clinical conditions will have to await the collection of additional data.

### Limitations and Priorities for Future Research

Co-occurrence rates among disorders represent important considerations in the development of risk models, societal cost estimates, theoretical accounts, and key aspects of clinical management. It is in the best interest of all stakeholders to have a clearer understanding of how often and under what circumstances language disorders and ADHD co-occur and what this entails for individual outcomes. Imprecisions, either at the level of our measurement systems or when brought in by overly flexible clinical constructs and taxonomies, undermine these efforts. In this report, data from a convenience sample initially designed to examine the accuracy of different language screening measures were repurposed to illustrate potential trade-offs in adopting different criteria for idiopathic language disorder. To this end, the data served their (re)purpose. The extent to which the estimates of co-occurrence rates calculated based on this convenience sample, however, would generalize to younger, older, more ethnically/racially diverse, or otherwise different groups of children is unknown. Estimates based on this convenience sample would need to be replicated in independent samples before placing too much stock in them. Arriving at replicable co-occurrence rates would represent a substantial improvement over the current situation, but it would be insufficient to guide clinical management. Unfortunately, co-occurrence rates tell us nothing about how comorbidity among disorders comes about within individual cases. Longitudinal studies following symptoms progression among children affected by idiopathic language disorder, ADHD, and both are needed to delineate the range of risk and protective mechanisms involved as well as their timing in the establishment of comorbidity.

Results of this study were limited by the measures selected. Parent ratings were used to estimate the severity of children's ADHD and S(P)CD symptoms. A key advantage to using standardized parent ratings is parents are uniquely situated to observe their children's behaviors across different contexts and over extended periods of time. A potential drawback of relying exclusively on parent ratings, however, is that observed overlaps in this convenience sample could have been a function of shared measurement variance. It is possible that reassessing the clinical intersections among idiopathic language disorder, S(P)CD, and ADHD using teacher ratings, self-reports, or basing them on children's performances on behavioral measures (e.g., continuous performance tasks, metapragmatic judgments) would yield different results. However, each of these alternatives involves their own psychometric limitations and trade-offs (Müller et al., 2012; Nyongesa et al., 2019; Whitcomb, 2018). The arrival of robust behavioral markers of S(P)CD would represent a welcome addition to clinical practice. In their review, Yuan and Dollaghan (2018) identified several candidate measures for capturing different aspects of S(P)CD symptoms that await empirical vetting to determine their diagnostic accuracy. Part of the vetting of potential clinical markers of pragmatic deficits should involve evaluation of their capacity to differentiate cases of S(P)CD from other clinical conditions, including autism spectrum disorder, ADHD, anxiety, ID, and idiopathic language disorder. Previous research on differentiating the psycholinguistic symptoms of SLI from the behavioral symptoms of ADHD provides a blueprint for how this work could proceed.

## Supplementary Material

10.1044/2020_JSLHR-20-00050SMS1 Transcript Transcript: 2019 ASHA Research Symposium: Sean M. Redmond, Clinical Intersections Among Specific Language Impairment, Social Pragmatic Communication Disorder, and Attention-Deficit-Hyperactivity Disorder

## Data Availability

2019 ASHA Research Symposium: Sean M. Redmond, Clinical Intersections Among Specific Language Impairment, Social Pragmatic Communication Disorder, and Attention-Deficit-Hyperactivity Disorder is available in the Figshare repository, https://doi.org/10.23641/asha.13063751.

## Footnotes

1 In the interest of full disclosure, I note here that I was one of the contributing discussants on the CATALISE Delphi exercise.

2
https://www.asha.org/Practice-Portal/Clinical-Topics/Spoken-Language-Disorders/Language-In--Brief/

3
http://pre.gcp.network/en/icd-11-guidelines/categories/disorder/developmental-language-disorder
