# Supplementary material for: Clinical Intersections Among Idiopathic Language Disorder, Social (Pragmatic) Communication Disorder, and Attention-Deficit/Hyperactivity Disorder
Source: J Speech Lang Hear Res. 2020 Oct 15;63(10):3263–76. doi: 10.1044/2020_JSLHR-20-00050 (PMC8363244; doi:10.1044/2020_JSLHR-20-00050)
Supplement: Transcript [file JSLHR-63-3263-s001.pdf]

Clinical Intersections Among Specific Language Impairment, Social (Pragmatic) Communication Disorder, & Attention-Deficit/Hyperactivity Disorder

So wow, that's a, it's a, um, quite an introduction. I'll try not to embarrass myself or Mabel (laugh) as I go, as I share with you some of the work we've been doing looking at… looking at the general issues of a differential diagnosis and comorbidity when it comes to two very common neural developmental disorders, specific language impairment and ADHD.

So as Mabel mentioned, the work that I'm sharing with you is, has been funded by NIDCD. And I realize I've committed a sin of not producing an acknowledgement slide, so I'll go ahead and acknowledge all of my colleagues and co-authors and collaborators, Andrea Ash, Kirsten Hannig, Amy Wilder, Tiffany Hogan, Kelly Christopulos, and Teresa Pfoff. Okay.

So, I've given enough of these talks and have made the error of not starting off with this note. To the effect that whenever I'm gonna be talking about children in our studies who have ADHD, some of them are on medication. And when they're on medication we ask parents to come in after washout. So these are children who are gonna be tested for their linguistic and nonverbal abilities unmedicated. It's also the case that when parents are asked to rate their child's behaviors in different dimensions, they're instructed to do so as if the child was not medicated.

Okay, and another quick note I need to make here is I started off interested in differential diagnosis of SLI and ADHD. And this was back in the early 2000s. And I thought I was perfectly lined up and ready to go. And as long as the taxonomy stayed stable, I was gonna be okay. (Some people laugh) But a couple of things happened along the way. So DSM5 came out, and then we also had some discussions about whether or not we should expand this characterization of kids with idiopathic language disorder to include some kids who haven't historically been included in studies of kids with specific language impairment. And so I'm gonna try and be consistent within myself, but maybe not with the rest of the field. And I'm gonna talk about thing, so that if you can think of this first line here as kinda like Russian nesting dolls. So the biggest doll is communication disorder, the one underneath that is speech-language impairment which is a catch-all term that's often used in the schools to characterize kids who hang out with speech-language pathologists. Then we have language impairment or language disorder, which would include kids who have impairments for all kinds of reasons, some of them are not necessarily developmental. Then we have developmental language disorder, which is a term that's getting used to describe kids in research studies in a specific language impairment. And the term "Developmental Language Disorder" as it's being used currently in the literature by many people, is just a synonym for SLI. And so they apply the same criteria as they would if they were talking about SLI. Now for the CATALISE

a consortium, a Delphi, uh, process that Dorothy Bishop had it, that was really not the intention of the term "Developmental Language Disorder." The idea was we would include kids who have specific language impairment as sort of classically defined by Stark and Tallal criteria, but it would also allow some kids who wouldn't of been included before who have low nonverbal IQ. Very low nonverbal IQ is where the threshold has been offered, that would also include kids who have ADHD, reading disability, behavioral disorders, and Social (Pragmatic) Communication Disorder, which was a new thing that came in with DSM5, which is why I'm struggling with how to integrate this in what I've been doing. It would be exclusive of intellectual disability and autism spectrum disorder. And then there's a caveat there about biomedical syndromes and conditions. And I would just point out at this point; that's redundant. Okay, so I would point out that, um… the, the term "biomedical" is, is… squishy, fluid, and it should characterize ADHD, because it's literally a condition that is treated with medicine. So that would make it a biomedical condition under the normal use of the term "biomedical," but is not that in this case.

So the DSM5 taxonomy, we have language disorder, and that would include kids that would fit most of what's going on there with the DLD from CATALISE. But it would exclude kids with Social (Pragmatic) Communication Disorder. Okay.

Then in the ICD11, which hasn't come out yet, but it looks like it's gonna shake out that we'll have receptive and expressive developmental language disorder, mainly expressive developmental language disorder, and mainly pragmatic developmental language disorder. And so this gets tricky when you're trying to talk about differential diagnosis and comorbidity. And so what I'm gonna do throughout my talk is kind of try to slide around in these different schemes.

Okay, so, we're gonna talk about in this presentation, specific language impairment in Social (Pragmatic) Communication Disorder. And we're gonna ask questions about how would we know that we have co-occurrence with ADHD that isn't artefactual or spurious, or due to some sort of conceptual construct problem.

Okay. And then another thing I need to talk about is, which is kind of highlighted when you, when you consider what's going on in the ADHD literature. When you use the term "disorder" in a research study, sometimes the kids who are being described have never been diagnosed with a disorder. They're just being, they're, that label is just being used to describe them. And so in the ADHD literature, you often have studies that are based on community samples, and they use the ADHD rating scale, and they identify some kind of cutoff as, as a child with elevated ADHD symptoms or not elevated A, ADHD symptoms. And, in a discussion about these two streams in the ADHD literature, where we have clinically diagnosed cases of ADHD, of children who've gone through the process of differential diagnosis, and have arrived at that classification, there are some sort, there's, there's some tradeoffs to both of these sorts of ways of looking at ADHD. So when you're looking with; when you're looking at clinically diagnosed cases of ADHD, you tend to get cases that are more severe than you would if you were collecting data and from the community sample. And you also have the interesting challenge of…

the, the interpretation of the results that you're collecting on kids with ADHD depend heavily upon your comparison group. Whether it's another clinical group, whether it's a group of kids who are typically developing, or whether it's a group of kids who are above average in things like nonverbal IQ and verbal abilities. And so one thing that I've noticed comparing our two literatures, is that the ADHD literature also has the phenomenon of comparison groups that are described as average or typically developing, and they're not so typical really. They have above average, more than one standard deviation above average performance in nonverbal IQ, which is a common occurrence in our literature as well. Kids with ADHD often have nonverbal IQ within that low average range.

So, with, with clinically diagnosed, you als—you, so you get this issue of spectrum bias where the more compromised kids are brought in. But you have to be careful about what you, what you're doing in the comparison groups. The; this is less of an issue in the community sample, but, it's, you're approaching it from a, a continuous dimension. But then you get into these weird squabbles about where the cutoff should be. And you also, um… are relying very heavily on the integrity of the informants that you're using. And the informants that are often used aren't sort of clinically trained to make those de, decisions, they're just filling out a, a questionnaire.

And so in, in syntheses of this literature, these two piles easily get mixed and kind of interpreted as, as sort of telling us the same thing. And I'm gonna be guilty of that today too. (A few people laugh)

Okay, so, I'm gonna talk about studies that I've done in sort of like both, uh, camps. So we've done some studies where we've recruited kids with ADHD because they had a diagnosis of ADHD, and the met our experimental criteria for ADHD. And then we compared them to kids with typical development, and kids with specific language impairment. But we also have some data that we collected recently; a community sample. And then this gives us the opportunity to bring in some kids who… may not have an ADH di dog—ADH diagnosis, but met criteria on the clinical informant rating scale that we're using.

And so from this perspective, I think we can look at our own literature and notice some similarities and differences. So I, I think it's safe to say that most of the literature that's been done on SLI has been similar to the, this first type of research in ADHD where we… we go into clinician caseloads, we try to find kids with language impairments. We can't go to the clinician and say "Give me all your kids with SLI," because they don't know what that means. If you say I want all your language cases, and you go, and you sift through them for your own research; so the kids haven't been diagnosed with SLI. That isn't really a diagnosis in the same way that ADHD is a diagnosis. And, we've also been squabbling a little bit about where the appropriate cutoff should be. Most notably, sort of somewhere between the zone of like a standard score of 85 or 80 on some kind of metric.

And in this presentation I'm talking about Social (Pragmatic) Communication Disorder. Nobody has been diagnosed with Social (Pragmatic) Communication Disorder. We collected this data before that was a thing. And (Laugh), we're gonna rely on the Children's Communication Checklist. And its pragmatic subscales to help us explore what's going on here between pragmatics and ADHD, and some other areas of language.

Okay, so that's what I'm gonna do is walk us through some old data reports, and then… there's some old data that's been repurposed for this talk.

Okay. So one way of, of asking, or thinking about co-occurrence or comorbidity, is… to wrap our heads around the difference between some symptoms that are clinical markers, or pathognomonic markers, or in the ICD system they're called essential features. And, symptoms that are call—that are referred to in the medical literature taxonomy says "assident." Now the term "assident" is defined by the urban dictionary as a collision between two people that were acting as asses. (Everyone laughs) But that's not, that's not the, that's not the… term (Laugh), that's not the definition I'm gonna use for this talk. So "assident" means these are symptoms that a clinician would encounter that are commonly associated with a diagnosis, but they're not necessarily indicative, they're not a requirement for that diagnosis. And within the category of "assident symptoms", we can think about, or we know of, that there exists transdiagnostics of, of symptoms. So transdiagnostic systems refer to the, the reality that there are some symptoms that appear across multiple clinical categories. And, that could be an interesting, or, it could be a relatively interesting or uninteresting observation. So, it, you could have purely descriptive transdiagnostic symptoms that really don't have any necessary or, or causal pathway, or mechanistic relationship to the disorders that it's associated with. So something like low self-esteem is a symptom that people could arrive at through many mechanisms. And those mechanisms don't have to have any meaningful relationship amongst them. Poor academics could be due for a lot a reasons. And if you encounter that in a case, it's not really helping you make the decision of what diagnosis to apply.

Now the more interesting transdiagnostic set of symptoms maybe are those that are potentially mechanistically transdiagnostic, meaning that they are setting up relationships across disorders due to sort of an underlying mechanism that, that creates a pathway to each of them. And so in the, in the area of, of psychiatry, uh, rumination, the inability to stop revisiting a, an emotionally charged event, is a core problem across all the emotional disorders. And, people have designed interventions around that idea that you could provide a common intervention to a class of disorders. So the, the, the goal, or the, or the hope for looking into tr—mechan, mechanistically, transdiagnostic symptoms, is that you'll be able to as a clinician, provide intervention to multiple categories, rather than providing intervention that's necessarily tailored to each one.

So yeah, you have path, pathognomonic and assident symptoms, and as a clinician, you have to work with both. And it's not the case that you would necessarily prioritize pathognomonic over assident symptoms. And in fact, a strong clinical marker would be one that we would expect under regular circumstances to be relatively resistant to treatment, if it's sort of capturing something that's essential about the individual's risk for

development. And as I mentioned, if we could imagine interventions, that are transdiagnostically aligned with how the mechanistic relationships across disorders work. That puts us in a, in a potent position for intervention.

So again, now going back into our literature, I think… in some ways, language disorders in general have been treated as descriptively transdiagnostic. And so you see this in the Bloom and Lahey scheme. ASHA's definition of a language disorder is sort of diagnostically agnostic. Maybe apathetic. (Some people laugh)

We, we also see this in, in the most common preprofessional textbook that we have. It's not uncommon for instructors that are directing a course in, on child language disorders to, to sort of bring across the message "ideology, smideology." If a child has a disorder in vocabulary, we're gonna treat it the same way, regardless, or whether they have autism or down syndrome, or SLI. This idea that you don't really need to fuss with diagnostic categories is, is… certainly a, a position that's been expressed.

And you also think that over the years when we've had a model suggested as a description of what's going on with, children with language disorders that have fallen back on general cognitive mechanisms like memory or executive function. They're really after the possibility that it might be a tran—the mechanistically tri—transdiagnostic symptom here that would link language disorders to other disorders. Now the challenge for things like executive function, as, as real—for realizing that goal has been that it's, it's… it's been very challenging to explain why two children with ADHD at the same level of severity, had very different executive function profiles. So one child will do poorly in executive function tasks, and one will do fine. And yet they both meet criteria if you had ADHD and both have the same level of severity. And it's even more challenging when you have to explain why an executive function deficit was responsible for… um… this child with SLI's language weakness is. And then you have a child with ADHD who's executive function is worse, but their language skills are within normal limits. And so this… hasn't been I think su—sufficiently realized yet.

So, I started to think about what would be, what would constitute evidence for a mechanistically transdiagnostic symptom, given the data I've collected. And, I think you, there's a couple of things that have to be demonstrated. So you have to show that a child with Disorder A, is doing worse than typically developing controls on a Disorder B measure. And vice versa. And, what happens is, we expect that the child with Disorder B, even though, even though they might not… reach clinical threshold, on that other clinical measure, they should sort of sit between the kid with, um, a di—with the other disorder, and the, and a group of typically developing kids. So, to put in the variables for A and B there, a child with… ADHD with, is Disorder B, and we should expect that their language abilities might be better than kids with specific language impairment. And, but worse than typically developing kids. And so that would sort of vindicate maybe a transdiagnostic mechanistically linked system here.

One thing that is almost axiomatic, in the psychiatric literature, is that… when comorbidity occurs, it always amplifies the symptoms in both conditions. And so if a

child, to put into, to terms we're gonna look at here, if a child has both ADHD and language impairment, we would expect, based on how it is played out with every other combination of disorders, that their ADHD symptoms should be worse in the child who has ADHD, but doesn't have a language impairment. And their language impairment should be worse than the child with specific language impairment who doesn't have ADHD. That is how comorbidity almost always works.

So, we want to… when we, when we explore comorbidity and co-occurrence, we want to focus on the pathognomonic symptoms. We'll leave the assident symptoms for the side, and focus on at least what's being offered in both areas as, so the clinical markers for language impairment and ADHD. And this also builds in an assumption that in terms of differential diagnosis, that both areas have built measurement systems that are robust enough to handle the task for differentiating between a atypical category. So most tests that we design take a look at whether or not they can identify a child who is typically developing from a child who is developing atypically. So a child who has normal language abilities versus a child who has atypical language, language disorder. But we rarely ask or bring in the criteria or the task that it should also be able to differentiate amongst non-language based disorders. So, for children with ADHD who don't have a language disorder, if a language measure seems to be penalizing children because they have ADHD, they can't complete the task, or, they get distracted, or, for some there's some, some timing element in the language task, then that's not necessarily a measure of their language abilities, it might be a measure of their problems with attention and impulsivity.

Okay, so, back in 2002 when I first started getting interested in this topic, I, I took a look at what's used to diagnose ADHD and other social-emotional dis—behavioral disorders. And, for the condition ADHD, there is no behavioral test. The diagnosis of ADHD is accomplished through, primarily through structured interviews with families, and through standardized informant rating scales. And, if you look at the content, or if you travel back in time with me to 2002, and you look at the content of, the scales that were being used to identify these behavioral disorders, there was a lot of language items. Literal language items on there, that are loading into identifying cases of social-emotional disorder. And, it was really bad in 2002. And so recently, we took a look at what he current situation is. And the current situation has had some improvement. One thing that's changed is some of these scales are offering disaggregated norms. So we have norms for children with learning disabilities. Some of them even have norms for children with language disorders available in their scales. Children with language disorders are being represented at higher rates, and they've started to incorporate some validity checks to make sure that you're not capturing information from an informant who's overly negative or overly positive. One of the realities of informant, uh, rating scales, is inter rater reliability is really low. There's, it's more often the case that parents and teachers are gonna disagree than agree. The cor—the correlation's like .3. However, it is true that if a parent says that my child has this problem, ADHD, and I'm putting them, I'm rating them in the clinical range, it's almost always the case that the teacher agrees with that. So the disagreement has been in teacher reported behavior problems that parents don't confirm.

One of the things that happened with the new DSM5 is that situational ADHD is not a thing anymore. You can't have an ADHD that only exists from 8:00 a.m. to 3:00 p.mo, (Laugh) it's absent on the weekends, and disappears over the summer. (A few people laugh) You have to have a confirming evidence, confirming symptoms in nonacademic settings. So that's nice. But if you, as you notice, the overlapping language academic items continue to be a problem. So these are some of the items that exist in some of the scales that we looked at. And so if you read through them, I think you might as a speech-language pathologist, consider that these could be a sign of a language disorder, maybe. At least, um.

Okay. So, um… one thing we did with the scales that were available at the time when we collected this data was, we looked at what happens on these ADHD rating scales if you took the language items out. And, your first reaction might be, oh no, you've broken the standardization. The scale won't work, you can't use it. But this is actually common practice in psychiatry, where we, where there's a concern for overlapping symptoms. ADHD as a construct seems to have a lot of overlap with other things like anxiety and sleep deprivation and things like that. So, we weren't, we weren't running rogue here. We were doing sort of like accepted best practice. And, what we found was that when you take the language and, and academic items out of the Child Behavior Checklist and the Connors, which are two of the most commonly used scales to identify ADHD, you don't break them, in fact, you improve them. You improve their capacity to differentiate ADHD from language impairment. And they're still just as good at identifying ADHD from typical development. So the scales don't need these items to be able to accomplish what they're after.

So, we had an opportunity to, to bring in a more current scale, more current version of the Connors, and, collect it on a, on a subgroup of kids. They all happened to be males. No, they didn't happen to be males, we selected them for a different project. (Laugh) But, for the dem—for demonstrating this little tidbit of information, the, the Connors has a negative impression index, a positive impression index, and then a consistence index. And this allows us to ask are parents of children with language impairments in denial? Are they just overly positive about their child's behaviors? And that would be picked up by these like validity checks. This is similar to what goes on in adult rating scales that catch malingerers on like the Mis—Minnesota. And what we found is in our two groups of language impaired kids and non language impaired kids; now I'm using that term because that language impaired group is gonna include kids that are more in the DLD group with a lot of different other things going on potentially. And then I don't say typically developing, I say non LI because that's gonna include some kids who are in other clinical groups, they just don't happen to have a language impairment. So they're gonna be some kids with ADHD, kids with behavior disorders in that group. Okay. See ow weird it gets when you try to like talk about these things?

Anyway, this isn't too weird. The… what we found was that there was overwhelming support for the idea that parents of kids with language impairments are not providing overly positive ratings.

Okay. So, when I started this line of research I, I honed in the SLI phenotype, which was emerging, and people were talking about nonword repetition, tense marking, and sentence recall, as a, a collection of things that might pan out as a clinical marker. And, we did a, a study looking at kids with ADHD that didn't have language impairment. Kids that were typically developing, kids that had specific language impairment. And, we did sensitivity specificity analyses, and the areas under the rock curve range from .88 to .96. And that was for both the, the SLI versus TD comparison, and the SLI versus ADHD comparison. So these, what this tells me is that if a child is ADHD, they're not necessarily being penalized for having ADHD on their performance here.

And since then, there was a, there was a very nice… scoping review or meta-analysis by Powlowska, that looked at multiple studies that were looking at keying in on the, on the 3 main clinical markers, and pointed out that our literature is compromised a little bit because we have spectrum bias, we have two gated recruitment design, where the clinical group is brought in because through clinical caseloads, and then the control group is recruited through the community. And, a one gated procedure is preferred, a community ample is preferred when you're doing these kinds of considerations of diagnostic integrity. It's also the case that our literature hasn't really embraced the idea of blinding during testing. And that's a valid criticism. And another observation that Powlowska made was that there's been inconsistent reference standards for what constitutes a hit, right? This kid has a language impairment because they met this criteria, but that criteria's been used in various research studies in various ways. So in our, in our recent community sampling project, we used blinding procedures, we used the community sample, and we also augmented the community sample with a, a, from a separate school district, a group of clinically recruited kids. We recruited kids that were receiving… speech-language services, kids who were regular ed, kids who were enrolled in emotional behavioral disorder programs. We excluded kids who were in gifted programs, and we excluded kids who were in ELL programs.

So, the main result that we found was that the tense marking and sentence recall screeners that we used turned out to be pretty good for a lot of the reference standards that we were considering. Okay. So this is a busy slide, but… we're gonna focus on the area under the curve. The interpretation of the area under the curve is taken as a, an overall estimate of the, of the instrument's diagnostic integrity. It is the probability that if you grab a kid from an, uh, the affected group, and a kid from the unaffected group, how likely is it that the kid in the effected group is gonna have a lower score. So, if it's .7, that's 70% of the time you're sorta like manually sampling from the two piles. Is as least one way of talking about the area under the curve. All of the areas under the curve are statistically significant at .001. The other cool thing about using ROC curves is you can identify the optimal cutoff. And the op—and, doing it this way is preferred over, over moving into this area with an a priori cutoff, that you believe has to be true. So it's not the case that a standard score of 85 or 80 is magically going to be the sweet spot for differentiating your groups. There's nothing that says it couldn't be 83 or 91 or 72. And what you can do with the optimal cutoff metric, the Youden's index, is identify where you get the best separation between your two groups.

So what we had in terms of reference standards was children that were just receiving speech-language pathology services, and we were able to confirm this with the school districts, that they were in fact; some parents didn't know that. And some parents were getting services outside the school. And so Dollaghan and Campbell have argued that that's like a ipso facto definition of language impairment. You're getting language services, so that means it's tapping into the functional impact of the language impairment, such that someone—it drew, it drew someone's attention enough to, to seek out services. So that makes it a bona fide language impairment. And similar arguments have been made for other clinical conditions too.

We looked at this Children's Communication Checklist as a way of also getting kind of the functional impact dimension brought in by a parent. Then we had the CELF-4, the TEGI, Dollaghan and Campbell's nonword repetition task. We went into the litera—we went into the discussion, the debate; should it be 85 or 80. And so we brought that in at different cutoffs. Then we had a composite which I think is, is aligns with how most people think of a language impairment criteria. That it should have multiple indicators. So if a child at least, had at least 2 of the following, they were identified as having a language impairment. And then we had a category where they had to have all of the clinical indicators. So this is gonna identify like the most severe cases of language impairment. And what you can ask with that is, does the cutoff that you use dramatically change, if we're going after a more severe or less severe reference standard.

So in terms of the sentence recall and tense marking measures, our weakest performance was with receiving speech-language pathology services. In the other areas we did quite well. So this is for sentence recall, and that, the, they're in the, in the low to mid 90s.

One thing I started to appreciate getting into the ADHD literature, is that… there's nothing like this level of diagnostic integrity. There's discussions in that literature that .7 is clinically adequate for area under the curve, and that anything above .8 is unrealistic.

So, I think that's pretty good. (Some people laugh) So this is past tense marking, and… there's a similar profile of performances, is doing reasonably well with some of our behavioral measures and the composites.

Okay, so… what's going on with speech-language pathology services as a reference standard? Why is that a potential problem?

So what I've done here is I've taken out two groups of kids in that study. One group of kids was identified only by virtue of receiving speech-language pathology services. None of the other measures were positive. This includes the parent report on the Children's Communication Checklist. There were 19 of these kids. And then we had a group of kids that were in that composite criteria who weren't receiving services, but they met at least 2 of the other ones. And, what we found is what other people have found when it comes to disparities in services. We found that there was, there tended to be more males in the served versus unserved group. They tended to be more white, but

that's generally true of Utah in general. We also had more concomitant conditions in the group of kids that were receiving services but didn't have another kind of language impairment. They also tended to be a little more affluent, and the two indicators that were statistically significant in our comparison here was mom's education level. If your mom had a college degree, you were more likely to be in the first group than the second group, and nonverbal IQ. So the nonverbal IQ of the kids receiving services, who don't have another kind of language impairment or, or met any other of the reference standards was 112, and then the untreated group was around the, the mean, 100. And so that's, that's why receiving clinical services might be limiting as a reference standard to aim our clinical measures at.

Here's a breakdown of those two groups in terms of, of the different concomitant conditions. And so, one thing to point out there is the, in the group of kids that are not receiving services but met at least 2 of the other criteria, the common, 70% of those kids don't have anything else going on. The majority of them would fit the definition, the classic definition of specific language impairment. Whereas, half the kids that were receiving services had that profile and half had something else going on, but it wasn't coming up as a language impairment with our language measures.

So, there's another group that is present in the untreated group that's not in the treated group. And that, and those were kids that had ADHD, and a behavioral disorder, receiving services for behavioral disorder, and a language disorder. So those kids were being overlooked. And that might have to do with diagnostic overshadowing. So, this kid has behavior problems, and everything that comes in is interpreted through the lens as a symptom of behavior problems, and not necessarily looked at for other things.

So, this isn't news to any of the other community samples that have looked at specific language impairment. If you define them experimentally, they tend to get overlooked for services. Most kids in a community level with specific language impairment are not on the caseload of speech-language pathologists, as we found here.

Some additional details. We didn't have a problem with inter-rater reliability, or test-retest ability. Administration times were brief. And you can, if you spend 2 hours with undergraduates, you can get them to fidelity.

Okay. So, at that point I think we've got reasonably good clinical markers of language impairment to start looking at going back to some a those mechanistically transdiagnostic assumptions that we started off with. So, we need to look at kids who have both the language impairment and ADHD, and compare them to kids who just have a language impairment. And what we found when we did that was there's no difference between kids with specific language impairment and kids who have a comorbid presentation. And in fact, there was a tendency for the comorbid kids to do a little bit better, which is always an unexpected outcome. We also found that there was no difference between our clinical cases and our community cases. We blended the two samples together to get enough comorbid kids. So this doesn't suggest mechanistically transdiagnostic relationships between these language measures and ADHD.

Okay, so our clinical markers of SLI that are in development, are robust for this challenge of differential diagnosis. There doesn't seem to be much evidence that there's… transdiagnostic overlap with ADHD. The measures of behavior do show overlap, but if you take them out, then you fix them. And so, if we wanted to study ADHD and SLI comorbidity, we would study kids who meet the criteria of having a deficit intense marking, nonword repetition, and/or sentence recall, and the ADHD symptomatology's been adjusted, this is primarily in the inattention domain for overlapping symptoms of language impairment. And then we would ask our questions of what was going on there.

Okay, so I've been juggling, ha, ha, ha, um… SLI and ADHD, and now I'm throwing in a third ball, Social (Pragmatic) Communication Disorder. This is the DSM criteria for Social (Pragmatic) Communication Disorder. And you'll notice that I've set up like most of this, uh, the DMS scheme. When this came on, there were questions about what this actually is or was. So one interpretation of this is this is just kids who didn't meet criteria on the ADOF, or the ADIR, and it's just a way for people to study the leftovers from research looking at autism. Is this high functioning autism or Asperger's just relabeled, is another possibility that people talked about. And, is this what we used to call pragmatic language impairment, which if you go back into our literature some, sometimes included kids with autism, and then sometimes didn't. Sometimes included kids with SLI and sometimes didn't. And, based on the social—I don't know what to do with that parentheses. Am I s'posed to whisper it? Social. Communication Disorder. I don't know. (He and several audience members laugh.) Why those parentheses are there. That's like… like excuse me pragmatic, oh. (Several people laugh) Um, but, um… some of the things to, to highlight here, you have to, you, you have to meet all a those criteria, you can't just have 2 of the 4 or 3 of the 4. It can't be due to low vocabulary and grammar skill, which starts to sound like what we could characterize as SLI, and it can't be due to another mental disorder. And I'm just gonna suggest that maybe we wanna consider ADHD as an example mental disorder to consider here with Social (Pragmatic) Communication Disorder. We used the Children's Communication Checklist. It's been the main basis if research looking at what's been referred to as pragmatic language impairment over the last 20 or so years. If you look at how people are using the Children's Communication Checklist, there really is little consistency across research groups. People pick and choose which subtest they wanna use and call it the Pragmatic Composite, and we did that too. (Some people laugh) But what we did was a little bit more deliberate. So Geralyn Timler went through the items on the Children's Communication Checklist and identified those scales that contained items that matched the criteria that we just went over. And so it's the Pragmatic Composite 5 is what we have going on here. And in this study we were interested in potential gender differences, because there has been reports in the literature sometimes, some reports say there is a gender difference, and some reports say there isn't. And, we were also interested in how pragmatics would load relative to language measures, but also relative to psychiatric measures, the, the clinical measures on the Child Behavior Checklist. And what we found were two different factor solutions for, for boys and girls. So for boys, pragmatics loads onto the same factor that ADHD and other externalizing problems do. It does not load onto a language factor, and not onto an internalizing behavior problem. For girls,

the situation is more complicated. It does load onto a language factor, and it loads onto what would be characterized as the general behavior problems, where the externalizing and internalizing are differentiated.

Now, the, the CCC-2 does not have separate norms for boys and girls. But all clinical measures looking at ADHD or social-emotional behavioral disorders do. You always have gender specified norms to identify affected cases. And so this is suggesting that pragmatics, which is different from these other language measures, is behaving in a way that's more similar to psychiatric scales and less so to what lang—so we didn't see lang—we didn't see gender differences in our language measures, besides pragmatics.

Okay, so now, um… here's some fun stuff. So what I'm gonna do now is I, I scooped up all the cases of language disorder that could meet any one of those definitions that I just talked about in all the cases of ADHD, and ended up with a pile of 85 kids. Kids with autism and intellectual disability were excluded. And, I have some operational definitions for what was, what's gonna happen now. So, a case of language impairment for this next analysis is gonna be characterized as performance on the CELF-4 core language scale, or yeah, core language score below 85. Ni=-- uh, nonverbal below 80, Child Behavior Checklist above 65, and, uh, we have… converted the, uh, pragmatic composite 5 and the standard scores, and we set the threshold at 80. And then what I'm gonna do is I'm gonna take a look at what happened when you apply these different taxonomies. And ask the question how much overlap is there between ADHD and these different operational definitions of language disorder. One that's advocated by the Catali—CATALISE, Cata, CATALISE, the Delphi report. So this would be cases of specific language impairment, nonspecific language impairment, and Social (Pragmatic) Communication Disorder. That was a very deliberate decision in that working group to incorporate pragmatics as a kind of language disorder, developmental language disorder. In the DSM scheme we would have SLI and NLI, but we would exclude S(P)CD. If you focus on the SLI operational definition, we don't typically exclude kids in our study samples of SLI because they have a pragmatic problem; that hasn't really been a thing. So I'm operationally defining this as plus or—you have you have, meet the SLI profile, and you may or may not also meet criteria on S(P)CD. And then we looked at just S(P)CD on its own. And, whether you agree with this scheming or not, or if you; or my interpretation of these diagnostic frameworks, there's a deliberate sort of like trickling down or filtering process going on which I think is at least interesting to look at. Okay.

So here we have 85 children that met the DLD definition, and ADHD. And you can see that… the way you're, I intended you to interpret this are, is that you would sort of glom the, the… I, I'm colorblind; why did I do this? Okay, so, (Several people laugh) the darkish orangey thing, and the blue thing together, and that would sort of give you a relative proportion with Developmental Language Disorder and comorbidity. And then you would go over to ADHD and Developmental Language Disorder and ADHD, and you would see for example that there's greater representation of comorbidity or co-occurrence from the ADHD side than from the language disorder side. Does everyone share my interpreta—Okay good. That's what I'm gonna stick with for the rest of the presentation. Okay. So, quite a bit of overlap there. If you, if you're interested in what's

sitting in that pile of kids with DLD, most of them have specific language impairment. A much smaller number of them have nonspecific language impairment. And there's a semi healthy representation of kids that just have Social (Pragmatic) Communication Disorder.

When we did the, we applied the DSM scheme, we get very little overlap. And most taxonomic systems do not want a lot of overlap, so this is progress. And then when you, when you bring in the possibility of plus or minus S(P)CD and SLI, you get something in the middle. Again there, it seems to be the case that there's a lot more overlap on the ADHD side with language than vice versa.

Okay, and then this is the last slide sort of like kind of demonstrating for the third time what I've already suggested, is that this overlap with ADHD is really a function of pragmatics. It doesn't have anything apparently to do with like what would be brought in from tense marking or non-repetition or sentence recall that might overlap with verbal working memory problems that ADHD kids might or might not have. Okay. So rate of overlap changed from 2 to 22%, that's a 10 fold increase. And this suggests to me that it matters when you're asking questions about overlap which taxonomy you're embracing. And you're gonna end up with variable results. And I predict a reproducibility is gonna happen. Non replication. It's good for keeping this employed, but not so good for answering questions about co-occurrence and comorbidity.

Now, admittedly, I only used the Children's Communication Checklist too. And I used it for something that it wasn't built to do. It wasn't built to diagnose S(P)CD. And, another measure of S(P)CD might function differently. And the problem is there isn't a diagnostic test for S(P)CD, so I can't bring that in to, to compare. Another possibility is when we think about what we're talking about with pragmatics. There might be constructural, is that a term; construct overlap between what people mean by ADHD and what we, we mean by pragmatics. And until we figure that out, coming up with a test is a waste of effort, because we don't know what we want, and we can't tell that we got there when we don't know what we wanted to find out in the first place.

So, here are some observations about how ADHD can be described in pragmatic terms. So a child with ADHD in a conversational context is gonna interrupt conversations, talk loudly, answer before the completion of questions, have excessive talking, and inappropriate vocalizations. And you don't have to invoke any underlying social cognitive deficits to have those symptoms come out. That's just a natural consequence of being impulsive. Same thing with the inattention dimension. If you're unable to carry on a conversation 'cause you're not paying attention to it, and you're answering the wrong question, the question you thought you heard, but not the one that you, was actually used, then you're also in the world of speech-language pathology, pragmatically disordered. So, (Laugh) until we can like articulate the boundaries, it's gonna be a circular argument. The other problem with Social (Pragmatic) Communication Disorder, is that it really isn't attached to a theory of pragmatics that would be driving the, the search for clinical markers, or the search for an underlying phenotype. You do have theories about pragmatic disorder. Michael Perkins has probably taken this the furthest. And he's

suggested an emergentist, non-modular theory of pragmatics. So pragmatics is not a module of language, it is the emergent property of the other language domains being implemented in real conversational service. And one of his predictions is that any disruption in any cognitive ability, perceptual domain, or social development, will inevitably create a, a pragmatic disorder. So, from that perspective, every chapter of the DSM5, is a pragmatic disorder, because it will result in conversational challenges. And when we're talking about pragmatics in the field of speech-language pathology, are we really talking very generally loosely about like something like social skills, or interpersonal misery? Is that what we mean by pragmatics, or is there something more concrete at stake here? So is pragmatics then our transdiagnostic clinical symptom, that doesn't really… require or necessarily need to have its own diagnostic category or clinical entity?

Okay, so, this is my last slide. One thing I think we could do to sort of maybe test some predictions at this moment. So it is the case that if you look at those ADH, ADHD descriptors, and you would take them over to the CCC-2, they're gonna load on… the coherence and initiation subskills. So one thing you could do is just toss those out and focus on the other ones, maybe. Another thing you could do is, if you wanna know whether ADHD was contributing to this symptom of, of pragmatic weaknesses, is if it's mechanistically linked to the ADHD symptom, then they should get better under medication, because the impulsivity is being managed a little, a little bit better. If it's an underlying social cognitive deficit, medication shouldn't have any effect on that. And I don't want to have the take home message be that, we're only interested in clinical markers when we're talking about research in specific language impairment or DLD, and, or, as a field we should only focus on the pathognomonic clinical features of our, of speech and language disorders, because of assident symptoms; remember the two asses colliding in, in their cars. (Laugh) those might actually, and, and for individual child, need to be where intervention needs to go. Because those are gonna be the things that are going to contribute or inform our characterizations of severity, and then they're certainly gonna help us operationalize something like functional impact. So, that's it. (Applause)

MARGARET: We'll have time for probably 3 questions which, I'm sorry, we're going to take from our research mentoring pair travel ward, if you know the drill. (Laugh). But for the rest of you that's just joined us, the NIDCD mentor protégé pairs have prepared questions. We will have a panel presentation coming up where we'll be able to have questions from everybody, so.

Q: Hi Dr. Redmond, thank you for your talk. I'm Caroline Larson, I'm a doctoral candidate from University of Wisconsin-Madison, I'm here with Klara Marton. So it was interesting to me that coherence popped out as ADHD symptoms loading onto that subscale. I've heard other arguments that coherence is more of a language task, where it draws on more structural linguistic skills. I was wondering if you could commle—comment on that and just…

SEAN REDMOND: So I . . .

Q: Provide some information about what coherence is, what it's measuring on that.

SEAN REDMOND: So sorry, I think where the… the… consideration needs to go is we need to, we need to do what I did with those… psychiatric scales and look for possible overlapping symptoms with language impairment, and flip that and do that with our rating scales of pragmatic competence. And then what you would do is you would do what we did with the, the Connors and the CBCL. You take them out and see if they still work. So I could imagine a study where you, you operationally define what S(P)CD is, you had a, you, you met all this criteria that we had, maybe even had like an official diagnosis… in play, and then you asked, does the CCC-2 effectively separate that group from a group of kids with ADHD, that don't have, don't meet criteria for S(P)CD. And to the extent that you can pull that off would be, would be, would; then you would be able to ask you know, what's going on here in other areas. Thanks.

Q: 'Kay, thank you.

Q: Hello, I'm Gabby Reynolds from the… University of South Carolina, here with Krystal Werfel. And you talked a little bit about the benefits of using a parent questionnaire, versus a teacher questionnaire. But I was wondering if you would anticipate any differences using a child questionnaire.

SEAN REDMOND: A what questionnaire?

Q: Uh, so where the child themselves fills it out, filled out the questionnaire.

SEAN REDMOND: Oh, oh. So, um, you could. Um… One, one, one of the… one of the limitations is, you have, you have to wait till the children are, are old enough to… adequately go, adequately complete these things. You can get, you can get feedback of— One of the problems is that, is a lot a kids will report that everything is just fine, right, even though their lives are falling apart. And that takes a while. It's a developmental phenomenon. So younger kids… don't have much to complain about, and as you get older, you start to complain more and more until you… are 52 like me, and you can't last 5 minutes without complaining. (Some people laugh) The other problem is, is that some of the scales that have been developed for things like personality and temperament are, are crazily complex tasks from a linguistic perspective. So one of my favorite example sis, this is, it would just be a, a therapist to a child; "What do you think your friends would say if they knew that you answered that last question the way you did?" (Some people laugh) There's a lot of that. That, that's an extreme example, but that, there's a lot of that going on. So you would need to like select them for, for grammatical accessibility. Now when you started your question, I was anticipating you asking a different informant, and that would be the teacher. And, some of the reasons why I'm hesitant to ge—to… go there is, well, one, at least in, in where I work, we're not allowed to pester teachers at all, so we can't collect informant scales. And when, when I; a long time ago, back in the late '90s where we bothered some teachers and got rating scales from them, we found out that they were reporting that in both groups of kids with SLI and typical development, about 30% of them were in the clinical range. So kinde—ki—

according to kindergarten teachers, they're all clinically compromised. (Some people augh) And then the other thing we found when we followed them from kindergarten to 2nd grade, is that, um… the kindergarten teacher would report the kid was in the clinical range, the 1st grade teacher would say they're fine, and then the 2nd grade teacher would say they're in the clinical range. There was very little consistency even between teachers. Meanwhile, parents were consistent across the, all 3 years of the project. So there's more stability in parent ratings. The oth—the other thing that happens when you try to do teacher ratings is, if you want… well you couldn't do for a community sample, because then you're asking a teacher to rate every kid in their class, and that's gonna break the system. (Laugh) So, and then there's that observation about the situational ADHD. So knowing that the teacher says the child's in the clinical range, isn't as informative as knowing that the parent put the child in the clinical range. And some people argue that the parent's rating scale is sufficient. You don't need to collect information from teachers. Now that's not according to best practices and, you know, that's kind of like an, uh, extreme view that's out there. But, it's supported by the, by that lack of relation—uh, inter rater reliability.

Q: Thank you.

Q: Hi, I'm Nat—I'm Natalie Pak. I'm here, I'm a doctoral student at Vanderbilt. I'm here with Dr. Kaiser. And my question is kind of related to what, what you were just talking about. I was wondering if you could speak to… kind of generally how… possibly the, the difficulty that we're having operationalizing social pragmatic symptoms, and how to characterize that could be affected by cultural differences between groups. If in the future you expect that to continue being an issue that we're dealing with. And I was also thinking as you were just talking, if you think that might have something to do with that instability across teachers, say if they have different norms that they're working from than maybe the child that they're, that they're rating.

SEAN REDMOND: Yeah. So I mean you could take, we could take lessons from the, our colleagues in psychology and… develop scales that are culturally… not, not… translated, but like completely re-normed within different communities. And so something like the Child Behavior Checklist has been re-normed in multiple languages and cultures. And there are vari—there are variations in, in interpretations of what social withdrawal represents for example. Some, some cultures that's an expected norm of behavior. And in our culture if you're an introvert you're, there's something seriously wrong with you, 'cause we sort of value the extrovert temperament over the introverted temperament. And yeah, you're right, it could be culture, even like more specifically brought down to the individual teachers having different value systems.

Q: (Inaudible comment)

Yeah.
